# Realized niche shift associated with the Eurasian charophyte *Nitellopsis obtusa* becoming invasive in North America

**DOI:** 10.1038/srep29037

**Published:** 2016-07-01

**Authors:** Luis E. Escobar, Huijie Qiao, Nicholas B. D. Phelps, Carli K. Wagner, Daniel J. Larkin

**Affiliations:** 1 Minnesota Aquatic Invasive Species Research Center, University of Minnesota, St. Paul, MN 55108 USA; 2 Veterinary Population Medicine, College of Veterinary Medicine, University of Minnesota, St. Paul, MN 55108 USA; 3 Key Laboratory of Animal Ecology and Conservation Biology, Institute of Zoology, Chinese Academy of Sciences, Beijing, China; 4 Department of Fisheries, Wildlife, and Conservation Biology, University of Minnesota, St. Paul, MN 55108 USA

## Abstract

*Nitellopsis obtusa* (starry stonewort) is a dioecious green alga native to Europe and Asia that has emerged as an aquatic invasive species in North America. *Nitellopsis obtusa* is rare across large portions of its native range, but has spread rapidly in northern-tier lakes in the United States, where it can interfere with recreation and may displace native species. Little is known about the invasion ecology of *N. obtusa*, making it difficult to forecast future expansion. Using ecological niche modeling we investigated environmental variables associated with invasion risk. We used species records, climate data, and remotely sensed environmental variables to characterize the species’ multidimensional distribution. We found that *N. obtusa* is exploiting novel ecological niche space in its introduced range, which may help explain its invasiveness. While the fundamental niche of *N. obtusa* may be stable, there appears to have been a shift in its realized niche associated with invasion in North America. Large portions of the United States are predicted to constitute highly suitable habitat for *N. obtusa*. Our results can inform early detection and rapid response efforts targeting *N. obtusa* and provide testable estimates of the physiological tolerances of this species as a baseline for future empirical research.

Understanding how certain species experience great success outside of their native ranges, often becoming more ecologically dominant than their performance as native species would suggest^1^ is a key challenge for invasion biology and has important implications for assessing risk associated with potential invaders. Examples of this phenomenon are numerous: Common reed (*Phragmites australis*) has suffered diebacks in Europe^2^, even as Eurasian genotypes have expanded throughout North America^3^. Monterey pine (*Pinus radiata*) has been reduced to five native populations in California, United States (U.S.) and Baja California, Mexico^4^, while being highly invasive in Chile, Australia, and New Zealand^5^. House sparrows (*Passer domesticus*) are extraordinarily successful as an introduced species despite declining in their native range^6^. Several mechanisms may drive these changes in fortune, including escape from natural enemies, altered population genetic structure, intra- and inter specific hybridization, novel allelopathic weapons, and unexploited resources^1^,^7^,^8^,^9^,^10^

Regardless of the underlying mechanisms, the success of some invasive species is attributable to their ability to occupy an ecological niche in their introduced range that is broader than or distinct from the niche realized in their native range^11^. It is true that many invasive species occupy niches very similar to those in their native ranges^12^, but for others an expanded realized niche leads to greater dominance within communities^1^, colonization of new types of habitats^13^, or growth under novel climatic conditions^14^. The gap between the realized niche in a species’ native range and its potential niche in a new range makes risk assessment more difficult, as even rare species can potentially become dominant under the right confluence of climatic, landscape, and biotic conditions^11^,^15^,^16^.

## *Nitellopsis Obtusa* Invasion in North America

A recent example of a largely rare native species becoming an aggressive invasive species is the spread of *Nitellopsis obtusa* (N.A. Desvaux) J. Groves (starry stonewort) in North America. *Nitellopsis obtusa* is a dioecious green alga in the Characeae family that is uncommon across much of its native range in Europe and Asia^17^,^18^ and is classified as a priority conservation species in the United Kingdom^19^, near threatened in Switzerland^20^, and endangered in Japan^18^, though there is evidence of expanded distribution in parts of Europe over the past few decades^21^. It occurs in shallow, fresh to brackish water at depths up to 10 m and can reproduce asexually via fragments and star-shaped structures called bulbils^17^. *Nitellopsis obtusa* was first found in North America in the St. Lawrence River in 1978^22^; it is now widespread in Michigan, increasingly common in New York and, since 2012, has been recorded for the first time in Indiana, Wisconsin, and Minnesota^17^,^23^.

### Detection, impacts, and management

*Nitellopsis obtusa* is of increasing concern in the Great Lakes region of North America. It appears to spread readily via human-assisted movement of fragments and bulbils (only males have been found in North America to date, precluding sexual reproduction), with occurrences associated with boat accesses and high-use areas^17^. Where it invades, *N. obtusa* can spread rapidly, grow tall and dense, and form surface mats, interfering with boating and recreation and potentially displacing native plant species^17^,^24^. Where *N. obtusa* does invade, effective treatment can be difficult to achieve. Manual removal may leave behind fragments and bulbils that can lead to reinvasion^25^. Currently available chemical control methods have been subject to little rigorous testing, and anecdotal reports from herbidice applicators indicate that treatments can result in a “haircut” effect, with upper portions of plants killed but lower portions intact and able to resprout^24^.

Challenges detecting *N. obtusa* and treating infestations compound the problem of its invasiveness. Charophytes are a taxonomically complex group and it can be difficult for non-experts to distinguish *N. obtusa* from other closely related, native muskgrasses and stoneworts (*Chara* and *Nitella* spp)^26^. Thus, it is possible that populations that are already established have not yet been detected. For example, when *N. obtusa* was first recorded in a Minnesota lake system in the summer of 2015, it was already present in an area >100 ha^27^, suggesting that it may have established years prior to being identified. Sleith *et al*.^17^ used a spatially stratified design to search for *N. obtusa* throughout New York State and found 18 previously unknown occurrences in a single field season.

### Potential distribution

**Figure 1:**
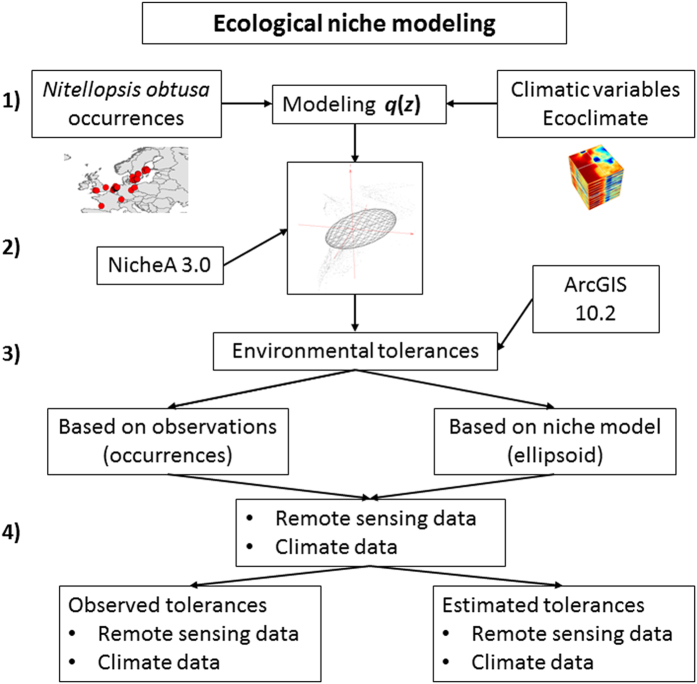
Framework to estimate the species’ niche, the potential distribution, and the environmental tolerances. (**1**) *Nitellopsis obtusa* occurrences and scenopoetic variables at coarse scale were collected. (**2**) An ecological niche model based on occurrences and climate data was developed as a proxy of the species fundamental niche. (**3**) Raw occurrences and the niche estimated based on a minimum-volume ellipsoid were used to identify the range of environmental conditions wherein the species can occur based on observations and niche estimation respectively. (**4**) The environmental ranges were estimated using both climate data at coarse spatial resolution and remote sensing data at fine resolution. This figure was generated using ArcGIS 10.2 (ESRI, Redland, CA; www.esri.com) and NicheA 3.0 (Qiao, H. *et al*.^67^. NicheA: Creating Virtual Species and Ecological Niches in Multivariate Environmental Scenarios. Ecography: 10.1111/ecog.01961; http://nichea.sourceforge.net/).

In light of the invasiveness of *N. obtusa*, uncertainty regarding its full distribution and physiological tolerance, and the limited toolkit available for its control, risk assessment to support prevention efforts is urgently needed. We performed ecological niche modeling to geographically evaluate invasion risk associated with *N. obtusa* and to investigate environmental conditions associated with its spread. Our approach is grounded in Hutchinson’s framework that a species’ niche comprises the confluence of suitable “scenopoetic” and “binomic” (biotic) factors^28^. In our niche model of *N. obtusa*, we focused on scenopoetic variables, defined as those abiotic environmental variables not consumed by the species and for which there is no competition among species^28^,^29^. Scenopoetic climatic variables, which operate at large spatial scales, are a robust source of information for characterizing multidimensional environmental space to estimate species’ fundamental niches, and have the advantage of being stable even when species’ abundances change^30^. Scenopoetic variables also help to define biomes, and are thus key components of species’ biogeography^30^. We estimated the niche of *N. obtusa* based on scenopoetic variables associated with its global occurrences. Our goals were to: (1) determine whether *N. obtusa* was exploiting novel ecological niche space in its invaded range, (2) predict its potential for further expansion in North America, (3) identify priority regions for early detection and rapid response efforts targeting *N. obtusa*, and (4) estimate the physiological tolerances of the species as a baseline for future research. Our first three goals were addressed using occurrence records from the native and introduced ranges of *N. obtusa* coupled with climatic variables. We used these data to generate a binary (suitable/unsuitable) niche model of *N. obtusa* as a proxy for the species’ fundamental niche. To estimate physiological tolerances (goal 4), we employed the binary ecological niche model and occurrence records as “masks” (i.e., spatial limits) to extract maximum and minimum values of climatic variables, and additional scenopoetic variables extracted from finer-scale, remotely sensed environmental data (Fig. 1).

## Results

**Figure 2:**
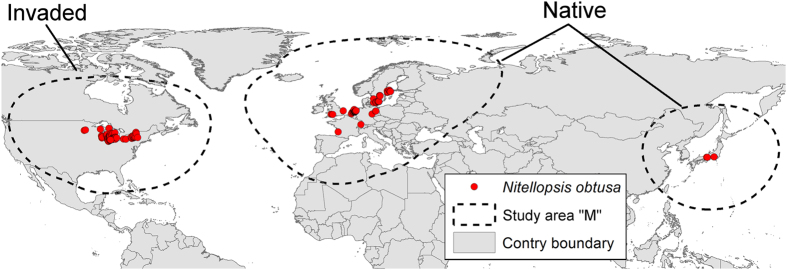
Study area and occurrences used in the ecological niche model of *Nitellopsis obtusa*. The model calibration areas, M, were estimated based on the maximum dispersal potential of the species in its largest geographic native range (Europe). We measured the maximum distance separating occurrences in Europe, resulting in a 2,150 km buffer; this distance (dashed line) was then applied across all available occurrences for the species (red points). This figure was generated using ArcGIS 10.2 (ESRI, Redland, CA; www.esri.com).

**Figure 3:**
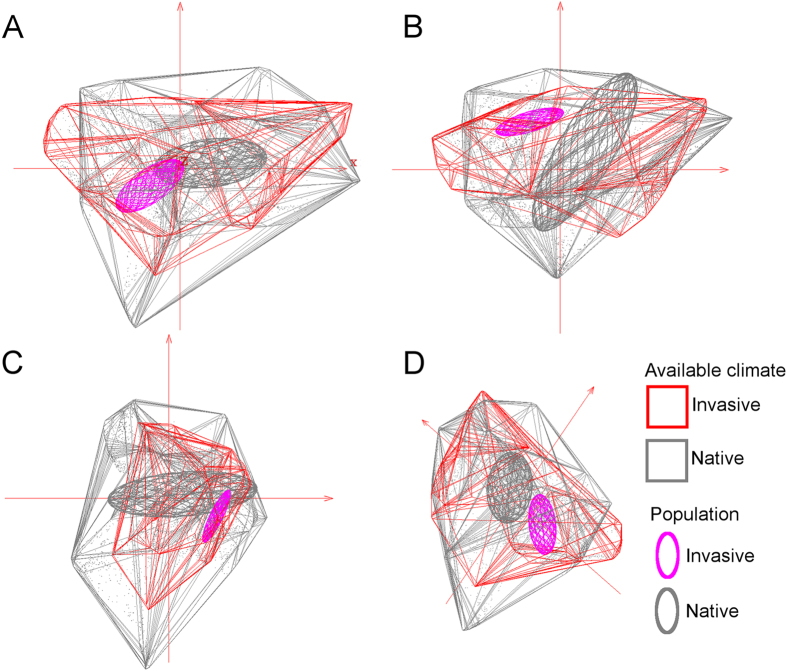
Native and invaded regions in (scenopoetic) environmental dimensions. Environmental conditions available in the native range (gray polyhedron) are compared with conditions available in the invaded range (red polyhedron). Environmental conditions under which *Nitellopsis obtusa* populations are found in the native range (gray ellipsoid) and the invaded range (pink ellipsoid) are also displayed. Visualizations of the: (**A**) first and second principal components (axes), (**B**) first and third principal components, (**C**) second and third principal components, and (**D**) three-dimensional visualization of the first three principal components. This figure was generated using NicheA 3.0 (Qiao, H. *et al*.^67^. NicheA: Creating Virtual Species and Ecological Niches in Multivariate Environmental Scenarios. Ecography: 10.1111/ecog.01961; http://nichea.sourceforge.net/).

We identified 2,255 occurrences for *N. obtusa* distributed across France (*n* = 1), Switzerland (1), the United Kingdom (5), Germany (7), Japan (46), Sweden (116), and the Netherlands (1,776), as well as the US (303; Supplementary Material S1). After removing duplicates, 846 unique occurrences were used for modeling the species’ native (Eurasia, *n* = 575) and invaded range (USA, *n* = 271; Fig. 2). Climate variables selected for model calibration included annual mean temperature, isothermality, minimum temperature of the coldest month, annual precipitation, precipitation seasonality, and precipitation of driest quarter. These variables were used because they represented the environmental information available throughout the entire study area and are likely to have biological significance for the species (Table 1). Using these climatic variables, we were able to generate a multivariate environmental space within which to estimate the ecological niche of the species for both native and invasive populations (Fig. 3).

**Figure 4:**
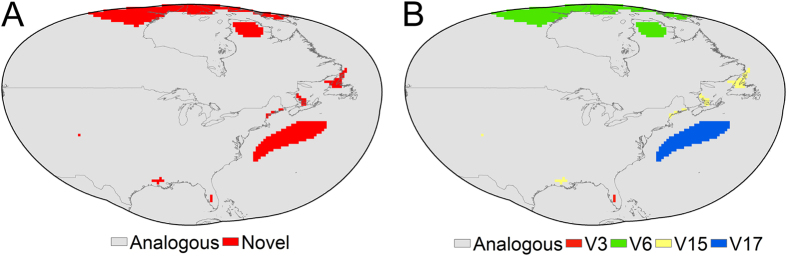
Exploration of novel environments in the invaded range. (**A**) Areas hosting novel environmental conditions not available in the native range (red) and analogous environments (gray) were identified. (**B**) Scenopoetic variables isothermality (V3; red), minimum temperature of coldest month (V6; green), precipitation seasonality (V15; yellow), and precipitation of driest quarter (V17; blue) were responsible of novel environments. This figure was generated in ArcGIS 10.2 (ESRI, Redland, CA; www.esri.com).

**Figure 5:**
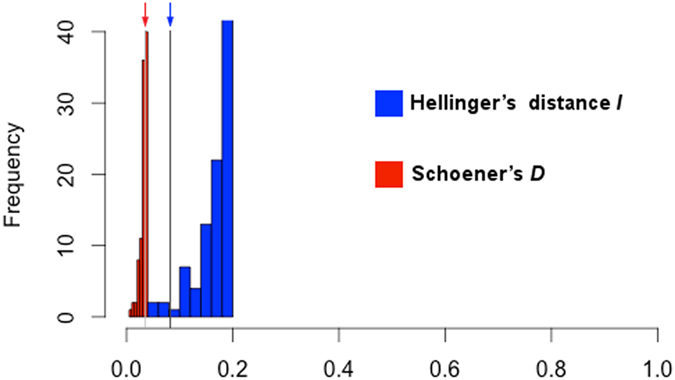
Background similarity test. Environmental conditions available in the native range and environments occupied by the species in the invaded range were compared using Hellinger’s distance *I* (blue) and Schoener’s D (red). Observed values (arrows) fall within expected values of similarity (null model distributions).

We found generally high overlap in environmental conditions available in the native and invaded ranges (Fig. 3). However, there was evidence of some “novel” (non-analogue) environments in the invaded region (Fig. 4). *Nitellopsis obtusa* occurrences in North America were not distributed within the same environmental space occupied in the native range. For example, there was no overlap between native and invaded ranges in terms of the environmental space occupied based on three non-correlated, multivariate environmental axes (Fig. 3). The novel climates in the invaded areas were identified in land and estuarine areas; variables shaping conditions distinct from those found in the native range included isothermality, minimum temperature of the coldest month, precipitation seasonality, and precipitation of the driest quarter (Fig. 4). To date, *N. obtusa* has not been recorded from these novel regions available in the invaded region. We were unable to reject the null hypothesis of similarity between the niche estimated in the invaded range and the environments available in the native range (p > 0.05; Fig. 5).

**Figure 6:**
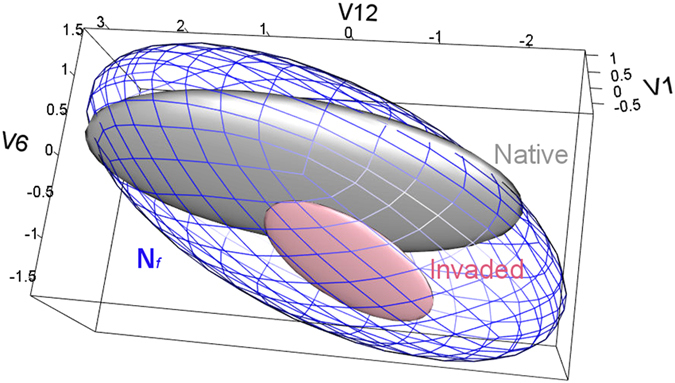
Ecological niche models for *Nitellopsis obtusa*. Models were estimated for the native (gray) and invaded (pink) populations, which resulted in non-overlapping niches. Thus, a final ecological niche model was generated by pooling all available occurrences (***N***_***f***_; open blue ellipsoid). These models were generated using variables V1, V3, V6, V12, V15 and V17 (see Table 1); this figure depicts environmental space based on three dimensions (V1, V6, and V12). Figure done using R^76^  (https://www.r-project.org).

**Figure 7:**
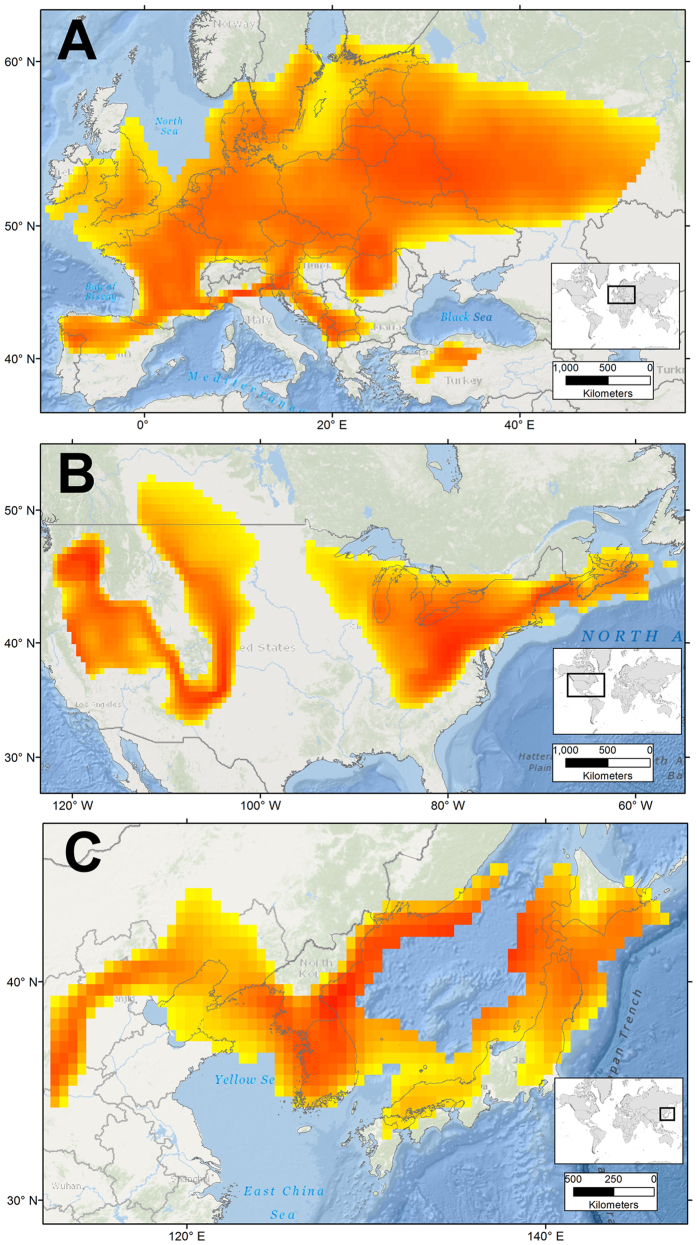
Geographically projected ecological niche model for *Nitellopsis obtusa*. Potential distribution of *N. obtusa* in coastal and inland waters in Europe (**A**), North America (**B**), and Japan (**C**). Shading is based on distance in multidimensional niche space to the niche centroid, and shows areas of relatively high (red) and low (yellow) environmental suitability restricted to coastal areas of 10-m water depth where the species is found. This figure was generated using ArcGIS 10.2 (ESRI, Redland, CA; www.esri.com).

Including occurrences from the invaded range expanded estimation of the fundamental niche of *N. obtusa*. The final model pooled native and invasive occurrences to estimate the species’ fundamental niche (Fig. 6, gray minimum-volume ellipsoid), with areas of potentially high environmental suitability identified based on distance to the niche centroid (Fig. 7). The ecological niche model predicted suitability in some regions with novel environmental conditions, these were concentrated on the Atlantic coast of the U.S. Highly suitable conditions were identified along the Sea of Japan and Peter the Great Gulf in Asia, throughout much of Eastern Europe, and, within the US, portions of the Eastern Temperate Forest, Great Plains, and Intermountain West ecological regions (Fig. 7). The fundamental niche estimated using scenopoetic climate variables was then used to quantify environmental tolerance ranges based on additional abiotic variables extracted from remotely sensed environmental data.

Environmental tolerances of *N. obtusa* inferred from known occurrences were narrower than model predictions. For example, we found that *N. obtusa* occurred in areas with annual mean temperatures of 4.96–14.21 °C, but our niche model predicted that it could occur at a broader temperature range (4.37–15.57 °C; Table 1 and Supplementary Material S2–S6). From our estimation of the environmental ranges based on fine-scale variables, we found that *N. obtusa* reports from coastal areas are characterized by dissolved oxygen of 5.72–8.33 ml/l, however, niche modeling values proposed tolerances as low as 4.95 ml/l, suggesting tolerance to more eutrhophic coastal habitats. Observed values for pH ranged from 8.18–8.24, with a mean of 8.2, similar to the mean value predicted by the model (8.18). Observed salinity ranged between 5.5–31.8 PSS, while the model estimated 3.8–38.4. Other fine-scale variables showed considerable differences between observed and modeled values of *N. obtusa* tolerance. For example, mean nitrate was 19.57 and 3.42 μmol/l for the observed and predicted values, respectively (Table 2). Mean land surface temperatures (LST) observed in inland freshwater systems range from 8–23 °C during daytime and 1–13 °C during nighttime. The niche model again predicted broader tolerances with mean LST of −5–33 and −8–16 °C during daytime and nighttime, respectively (Table 2).

## Discussion

### Main findings

We developed an ecological niche model for *N. obtusa* to assess its multidimensional climate tolerance and refined this information using biophysical variables derived from satellite imagery to characterize other environmental factors potentially associated with occurrence of this species. We then used the modeled niche of *N. obtusa* to predict which geographic areas likely contain environmental conditions suitable for this species. We found that, in its invaded range, *N. obtusa* is occupying environmental conditions not occupied in its native range (Fig. 3). However, a background similarity test showed that niche differentiation between the native and invaded ranges was not statistically significant.

### Environmental tolerances

The environmental range predicted for *N. obtusa* based on scenopoetic variables (Table 1; Supplementary Material S2–S6) provides a baseline for finer-grained observational and experimental investigations of the species’ biology. We found that minimum and maximum values of the scenopoetic climatic variables derived from the niche model were broader than the ranges observed based on locality information, suggesting *N. obtusa*’s potential expansion into new environments. For example, with respect to minimum temperature of the coldest month, occurrences correspond to a minimum temperature of −18.68 °C, but the model predicts that *N. obtusa* could occur in areas with temperatures as low as −20.11 °C, 1.4 °C below the minimum temperature observed to date (Table 1). However, this prediction was based on the assumption of a Gaussian response to climatic variables, which has been supported by results from other species^31^,^32^,^33^,^34^,^35^, but would need to be tested for *N. obtusa* specifically for robust validation.

Previous attempts to characterize the ecological niches of aquatic invasive species have generally focused on inland climate variables—even when focal species’ ranges have extended to coastal or marine environments, which may limit full recognition of potentially invadable environments^36^. Our results suggest that incorporating environmental information from both inland and coastal sources provides a richer representation of the species’ environmental niche. Integration of land and marine climate data in previous ecological niche models was limited by lack of availability of climate data layers covering both ecosystems. However, with the release of the Lima-Riberio *et al*.^37^ dataset, this is no longer a constraint.

### Realized niche shift

The presence of *N. obtusa* in broadly similar environments where it occurs as native or a non-native species suggests that its fundamental niche has been conserved during the invasion process in North America^38^,^39^. However, *N. obtusa* is using environments that, based on occurrence records we identified, are not occupied in its native range. This could arise due to human movement of *N. obtusa* to a new range, allowing it to overcome biogeographic barriers that constrained its potential distribution as a native species. Alternatively, *N. obtusa* may have expanded into new environments, occupying previously unfillied portions of its fundamental niche, as a result of release from natural enemies that may have limited its native range^30^,^40^. Occupancy of novel portions of a species’ fundamental niche in separate geographic regions is termed a “realized niche shift”^16^,^41^. A realized niche shift does not suggest evolutionary adaptation of a species to novel environmental conditions, but rather an expansion into portions of the fundamental niche that potentially could have been (but were not) occupied in the native range^16^,^42^. This finding allowed us to identify uninvaded areas throughout the U.S. that could be at risk of *N. obtusa* invasion in the future (Fig. 7) —areas that could not have been identified based on occurrences from its native range alone.

We found that environments occupied by *N. obtusa* in its invaded range did not fundamentally differ from environments available—though not necessarily occupied—in its native range (Figs 3 and 4). However, lack of environmental overlap between extant native and non-native populations was observed in multivariate environmental space (Fig. 3). Such dissimilarity may be imperceptible in geographic space (Fig. 2), which can limit understanding of invasion dynamics and the potential for future spread. Previous models of biological invasions have invoked evolutionary changes in species’ environmental tolerances to explain apparent fundamental niche shifts inferred based on models’ failure to predict invaded ranges using native range data (e.g.^14^,^43^,^44^). However, failure to accurately forecast invaded ranges may arise from stochastic differences in species’ environmental distributions that are not indicative of selection, and thus do not require niche evolution to be overcome^36^. In the present study, models of *N. obtusa* calibrated based on the native range alone would have failed to predict current occurrences of the species in North America due to non-analogous environmental conditions occupied by the species in the invaded range (Fig. 3).

### Potential for future expansion

There has been relatively little investigation of the ecology of *N. obtusa*, particularly in its invaded range. Novel environmental conditions exploited by *N. obtusa* in North America provide insight into the process of invasion. The patterns we observed suggest that there are gaps in environmental occupancy for this species in North America, i.e., the potential niche is not filled^42^. Thus, it appears that this species has not reached equilibrium in its ecological distribution. Invasion of new geographic locations and currently uoccupied portions of the fundamental niche are likely to occur as dispersal barriers are overcome by unintentional human movement. The rapid spread and robust growth of *N. obtusa* in the Great Lakes region suggests that environmental conditions within this landscape constitute highly suitable habitat, and our ecological niche model predicts other, as yet uninvaded, hotspots elsewhere in the U.S.

Of the 29 states in the U.S. that contain at least a small area of moderate to high predicted suitability for *N. obtusa*, only 5 have known occurrences to date: Michigan, New York, Wisconsin, Indiana, and Minnesota. This suggests that there is substantial risk of *N. obtusa* expansion in the U.S., with the species perhaps at an early stage of progression toward becoming more widespread and dominant^45^,^46^. Detailed field sampling to characterize conditions associated with *N. obtusa* populations and controlled experiments assessing the influence of environmental parameters on fitness are needed to empirically explore this species’ true environmental tolerance.

Prevention of further spread could be supported by early detection and rapid response efforts. Increased awareness of and research on *N. obtusa* in North America will hopefully result in aquatic plant monitoring, early detection, and management professionals being more likely to identify relatively new infestations, when control is more feasible^24^. Our maps suggest areas without known occurrences where surveillance might be especially valuable, particularly in Western and Mid-Atlantic States (Fig. 7).

Finally, one implication of our findings is that climate change could have a large influence on the future distribution of *N. obtusa*^47^. Occurrences in both the native and invaded range are concentrated in northern latitudes (Fig. 2), which are expected to be subject to large changes in temperature and precipitation^48^,^49^. Our findings indicate that these climate variables are important components of the ecological niche for *N. obtusa*. To refine *N. obtusa* risk assessment, a critical next step is to predict the influence of climate change on future geographic distribution of the species. Such an investigation might, for example, indicate greater risk for expansion in Minnesota and Wisconsin and lower risk in Mid-Atlantic states than we have predicted here.

### Methodological advances

Examination of both native and invasive populations in climate space expanded estimation of the niche of *N. obtusa*, enabling us to better approximate this species’ fundamental niche. Our results reinforce that niche models for assessing invasiveness should not be calibrated based on populations defined by administrative areas of interest^50^, instead models should be calibrated based on species’ entire ranges to capture the most complete environmental information available.

In North America, *N. obtusa* has apparently been spreading only by asexual means^17^, limiting genetic diversity of populations in the invaded range. Aggressive expansion of *N. obtusa* in the invaded range also contrasts with its rarity and conservation concern in much of its native range. The “niche centroid” hypothesis^51^ proposes that species’ populations that are nearest to the niche centroid (puatatively optimal environmental conditions) will have the highest population growth^52^ and genetic diversity^53^. Evaluating the validity of this prediction for invasive species will inform understanding of the true dimensions of invasive species’ niches, increasing fundamental biological understanding and supporting applied efforts to prevent further spread. *Nitellopsis obtusa* populations in the invaded range are occurring in a combination of climatic conditions not occupied in the native range, suggesting that dispersal limitation in the native range may be limiting filling of suitable portions of the niche. If the niche centroid hypothesis applies in the case of *N. obtusa*, populations closer to the niche centroid should have higher growth rates. This prediction requires empirical investigation.

Our model results should be viewed as baseline estimates of tolerance ranges for *N. obtusa*. Mean values of these ranges are approximations of conditions under which survival and growth should be high, i.e., environmental optima^52^. Alternatively, there may be biotic factors mediating *N. obtusa* invasion and population growth at finer scales that were not captured by our analysis. Competitive interactions with other macrophytes, depredation, and even pathogens or negative feedbacks with microbial communities may be more pronounced in the species’ native ranges^40^,^54^.

NicheA software added biological realism to our models by allowing us to: i) visualize the species distribution in environmental dimensions, ii) simulate the response of *N. obtusa* to environmental variables, and iii) predict invasion risk based on the niche centroid^52^,^55^,^56^. Areas predicted to be at high-risk based on environmental suitability were not clustered geographically, indicating the strong capacity of this approach to identify environmental suitability–relative to correlative methods that tend to interpret higher occurrence densities as necessarily indicating higher suitability, which can lead to spatial autocorrelation and model overfit^57^,^58^. This study prompted the development and release of new analytical tools: “*Generate Niches from Occurrences*” and “*Export Niche as Continuous Raster*”; these are now available within NicheA software 3.0 to facilitate the application of ecological niche modeling to predicting spread of other aquatic or terrestrial invasive species (http://nichea.sourceforge.net/).

### Issues of scale in modeling aquatic invasive species

Scientific literature on modeling the ecological niche of aquatic invasive species is scarce, perhaps because resource managers are often more interested in finer-scale forecasts pertaining to the regions they manage, or becaue waterbody-specific environmental variables are of great importance but can be difficult to obtain^50^. Managers often require fine-scale models explaining potential expansion of aquatic invasive species, even being interested in suitability estimations for specific microhabitats within individual waterbodies, modeling at such scales can be difficult (but see^59^). Species’ geographic distributions are the expression of complex interactions among abiotic tolerances, dispersal dynamics, and biotic interactions^60^. We limited our investigation to abiotic factors expected to shape *N. obtusa* current and potential distribution. Such coarser-scale, abiotic analyses for aquatic invasive species are critical for understanding biogeographic patterns of past invasions and for predicting areas at risk in the future^50^. Such analyses are a useful starting point for fine-grained modeling and empirical investigations.

## Methods

### Ecological niche modeling

We performed ecological niche modeling using an approach proposed by Drake^61^ termed “range bagging.” This is an ecological niche modeling approach that aims to characterize species’ abiotic tolerances in multivariate environmental space from geographic locations of the species. A challenge for niche modeling is reliance on presence-only data, given lack of availability of robust species absence data^30^. Correlative presence-only models are strongly influenced by the study area extent used for model calibration^62^. Ecological niche modeling using range bagging requires presence data from the species of interest and a set of environmental factors defined by the researcher; the method is not considerably influenced by the study area extent in delineating the ecological niche and does not require absence data. Range bagging assumes that niches are convex and simply connected in a multidimensional environmental scenario, providing biological realism to estimations and reducing the effects of sampling bias.

**Figure 8:**
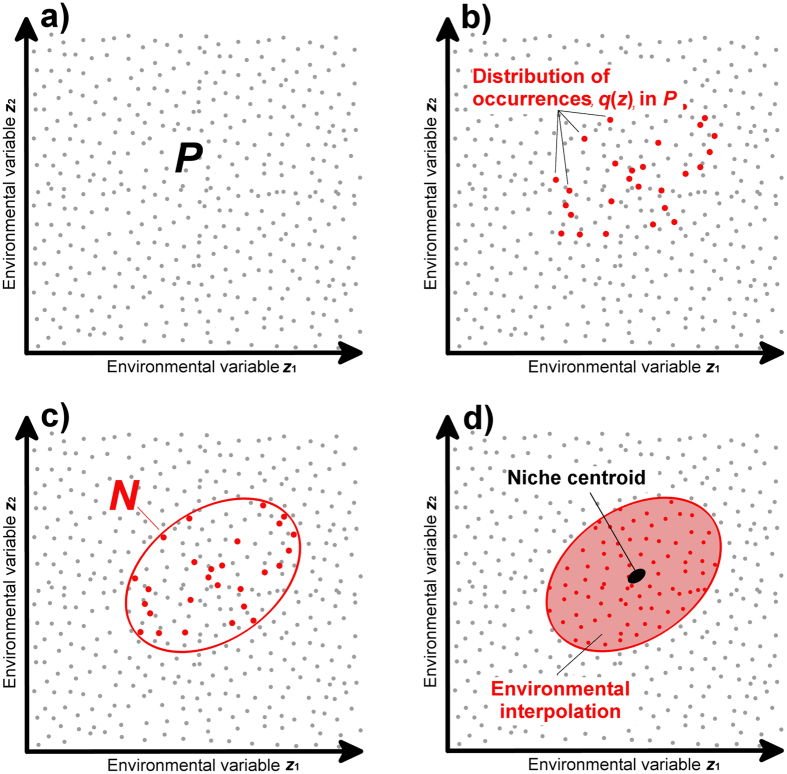
Ecological niche modeling framework. (**a**) Bivariate (*x* = 2) environmental space, *P*, constructed from environmental variables *z*_1_ and *z*_2_ (with values represented as gray points). (**b**). The distribution *q*(*z*) of the species’ occurrences *k* in the environmental space (red points). (**c**). Occurrences are used to build an existential niche model, *N* (red ellipsoid), as a proxy of the species fundamental niche, *N*_*f*_ (Drake^61^). (**d**). The niche model *N* uses interpolation of environmental values between occurrences (red areas within the ellipsoid). The niche centroid is estimated to identify the core of the niche, which is presumed to represent the most suitable environmental conditions.

The Drake^61^ approach characterizes a species’ multidimensional (*n*-dimensional) environmental space, ***P***, using *a priori* selected environmental variables, ***z***. The species’ range for each environmental variable, ***q***(***z***), is determined based on occurrence records, ***k***. Thus, ***q***(***z***) is the environmental distribution of occurrences ***k***, within environmental space ***P***. We assume that ***q***(***z***) is the set of environments in which the species’ population can persist without further immigration being required, i.e., “fundamental niche,” ***N***_***f***_^30^. Because occurrences may include both imperfect and incomplete sampling, ***q***(***z***) represents an approximation of ***N***—the “observable” or “existential” niche (*sensu* Peterson *et al*.^30^). Here we assumed that ***k*** **⊆** ***q***(***z***) = ***N*** **⊂** ***P***^61^. We estimated ***q***(***z***) separately for the native range (using native records, ***k***_n_) and introduced range (using ***k***_i_), to allow for the possibility that the realized niche would differ by range (Fig. 8).

Ecological theory proposes that niches have a Gaussian nature derived from species’ physiological tolerances to multivariate environmental conditions^31^,^35^,^61^,^63^,^64^. A species’ niche constitutes an *n-*dimensional “hypervolume” within a high-dimensional ecological space, i.e., ***z*** > 3 [ref. 65]. Along each dimension, species are likely to show a bell-shaped fitness response (normal distribution with the left and right tails and peak representing suboptimal and optimal conditions, respectively^31^,^35^,^61^,^63^,^64^). Given these patterns, an ellipsoid shape provides a simple and reasonable proxy of a species’ ***Nf ***61,66. This approach adds biological realism to estimates of species’ environmental tolerances and allows for interpolation along environments gradients, mitigating model overfit.

To perform this estimation using multiple environmental variables, we developed a novel tool “*Generate N(s) from occurrences*” which is now freely available in version 3.0 of the software NicheA^67^. NicheA generates a binary ecological niche model (suitable/unsuitable) via an environmental envelop algorithm that identifies space within a multi-dimensional environmental hypervolume occupied by occurrences of a given species. NicheA then generates a convex-polyhedron around all ***k***, allowing posterior estimation of minimum-volume ellipsoids circumscribing ***q***(***z***), as a proxy of the species’ niche. NicheA involves mapping occurrences into environmental space, such that occurrences that are geographically distinct may still share high environmental similarity.

Detail on the use of NicheA to generate ecological niches from species occurrences has been published elsewhere^66^, detailed description of this process can be found at http://nichea.sourceforge.net/function_create_g4.html. The environmental scenario to estimate the species’ niche was constructed based on scenopoetic (climatic) variables. We managed the ecological niche model as a climate envelope of ellipsoidal form. This provided a binary map of suitable (inside the ellipsoid) and unsuitable (outside the ellipsoid) climatic conditions. This model was then projected to the geographic space as a binary species distribution model. This binary model was then used as a mask (i.e., geographic delimitation of the niche) to extract the environmental values from remote sensing data (Fig. 1).

We developed models for the native and invaded ranges and a final binary model pooling occurrences from both ranges. In the binary model, we quantified the distance to the niche centroid by dividing the minimum-volume ellipsoid by 100 units from the Euclidean distance of the ellipsoid centroid to its edge—where the ellipsoid centroid is zero and areas furthest from the ellipsoid centroid are 100—yielding an index characterizing the range of niche suitability^66^. We considered areas closest to the niche centroid to be most suitable for the species’ population growth, abundance, and genetic diversity, based on prior empirical investigations of these relationships^52^,^53^,^55^,^68^. To perform this analysis, we developed the tool “*Export continuous ENM*,” which is now available in NicheA 3.0.

### Occurrences

Spatially referenced occurrence data were collected from herbarium databases accessed through the Global Biodiversity Information Facility^69^ and the Global Invasive Species Information Network^70^, using the keywords: “*Nitellopsis obtusa*”, “*Nitellopsis obtusa* var. *ulvoides*,” and “*Chara obtusa*”. Additional occurrences for the United States were collected from published sources^17^,^23^,^24^,^71^. Geographic coordinates (latitude and longitude in decimal degrees) were compared with reported localities to identify and remove inaccurate records, final coordinates were then revisited and duplicate records removed.

### Environmental variables

**Figure 9:**
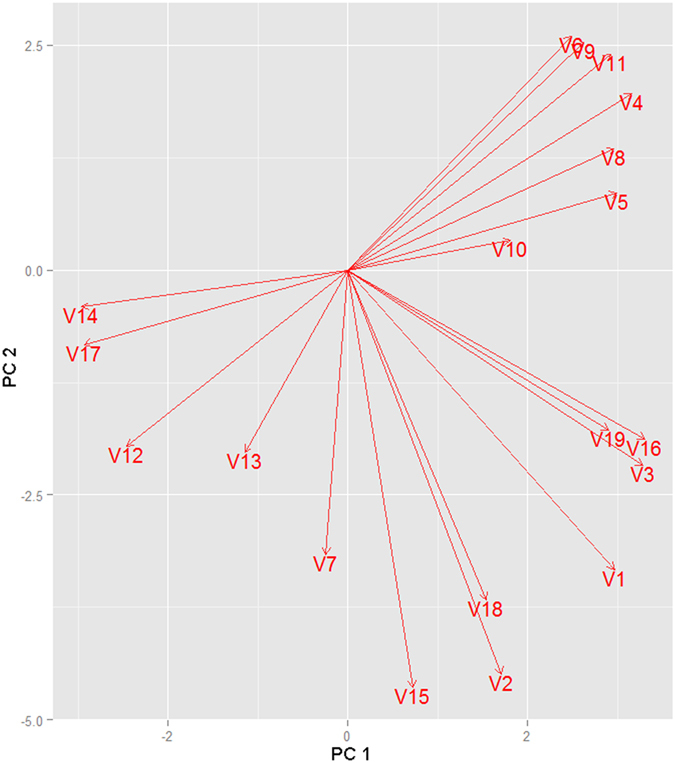
Principal components of the environmental variables used in the modeling process. Components are displayed in two dimensions, component one (PC1) and two (PC2), to show the association among variables. V1 = annual mean temperature; V2 = mean diurnal range; V3 = Isothermality; V4 = temperature seasonality; V5 = max temperature of warmest month; V6 = min temperature of coldest month; V7 = temperature annual range; V8 = mean temperature of wettest quarter; V9 = mean temperature of driest quarter; V10 = mean temperature of warmest quarter; V11 = mean temperature of coldest quarter; V12 = annual precipitation; V13 = precipitation of wettest month; V14 = precipitation of driest month; V15 = precipitation seasonality; V16 = precipitation of wettest quarter; V17 = precipitation of driest quarter; V18 = precipitation of warmest quarter; V19 = precipitation of coldest quarter.

Given the breadth of *N. obtusa* occurrences, i.e., that it is found in inland to coastal and freshwater to brackish habitats, we used bioclimatic environmental variables capturing patterns for both land and coastal ecosystems. Bioclimatic variables are a robust representation of scenopoetic variables^28^. We began with 19 climate variables that reflect long-term values of temperature and precipitation at ~50 km^2^ spatial resolution from the Ecoclimate repository^37^ available at http://www.ecoclimate.org/ (Tables 3). We evaluated collinearity among these variables via principal component analysis using the software NicheA 3.0 [ref. 66]. Collinearity between pairs of variables was examined using bi-dimensional vector plots. Where collinearity was found to be high, the variables comprising greater information content, i.e., covering a longer gradient, and with clearer biological bases, were retained and the other variables excluded (Fig. 9). This resulted in six climate variables being used in the final model (Table 1).

We performed hierarchical post-processing to determine species’ distribution in relation to other fine-scale environmental variables (Fig. 1). Briefly, the niche model developed using scenopoetic variables (i.e., climate) was employed to estimate *N. obtusa*’s niche. The resulting binary model was then used to extract values from all the climatic variables and also from remotely sensed environmental variables at ~9-km spatial resolution for coastal areas^72^ and at ~1-km resolution for inland regions^73^ (Table 4). Finally, we also used *N. obtusa* occurrences to extract the environmental values that it apparently tolerates under field conditions. Environmental values collected by occurrences were termed the “observed” environmental range and those derived from spatial masking of the binary ecological niche model were defined as the “modeled” environmental range (Tables 1 and 2). Predictions were constrained to areas <100 km off the coast to include brackish, coastal habitats up to 10 m water depth^17^,^74^. For niche model estimation, we developed the tool “*Occurrence statistics*,” which is now available in NicheA. Data management and analyses were performed using ArcGIS 10.2 [ref. 75], R 3.2.1 [ref. 76], and NicheA 3.0 [ref. 66].

### Study area

The extent of the geographic area considered influences ecological niche model outputs^62^; therefore, study area estimation should be based on the natural dispersal capacity of the species of interest^30^. We estimated dispersal distance using native populations in Europe, which are surrounded by biogeographic barriers (e.g., the North Atlantic Ocean and Tibetan Plateau) that separate them from other regions, including disjoint populations in Japan. We measured maximum distance separating occurrences in Europe as an indicator of intrinsic dispersal potential. This distance (2,150 km) was then used to generate a buffer around all occurrences. The resulting polygon constituting our study area was used to calibrated ecological niche models (**M** *sensu* Soberón & Peterson^60^; Fig. 2).

### Invasion process

The multivariate environmental distribution of *N. obtusa* was explored using the first three orthogonal principal components (axes) of a principal components analysis of the bioclimatic variables (Table 5). Populations and available environments in the native and invaded ranges were displayed using the software NicheA 3.0 [ref. 66]. Additionally, to compare native and invaded environments for the original scenopoetic variables, we used the multivariate statistical tool ExDet^77^. Finally, we tested a one-way niche similarity using the Schoener’s *D* and Hellinger’s distance *I* metrics for background similarity testing. These analyses were performed using ENMTools 1.4.4 [ref. 78]. These similarity tests evaluate whether the invasive niche is more similar to the native niche than expected by chance^79^.

## Additional Information

**How to cite this article**: Escobar, L. E. *et al*. Realized niche shift associated with the Eurasian charophyte Nitellopsis obtusa becoming invasive in North America. *Sci. Rep.* **6**, 29037; doi: 10.1038/srep29037 (2016).

## Supplementary Material

Supplementary Information

Supplementary Information

## Figures and Tables

**Table 1:** Environmental variables used for the final niche model for *Nitellopsis obtusa*.

Variable/Range	Observed	Modeled
Annual mean temperature, °C (V1)	4.96–14.21	4.37–15.57
Isothermality, % (V3)	15.51–42.02	7.73–44.81
Minimum temperature of coldest month, °C (V6)	−18.68–5.53	−20.11–9.63
Annual precipitation, mm/m^2^ (V12)	635.4–1819.69	396.66–1827.1
Precipitation seasonality, % (V15)	12.65–39.15	9.78–117.43
Precipitation of driest quarter, mm/m^2^ (V17)	88.56–296.04	15.79–422.45

Values based on known occurrences (observed) and those predicted by the ecological niche model (model).

**Table 2:** Description of the environmental range of *Nitellopsis obtusa* based on fine-scale environmental variables.

Coastal	Observed			Modeled			Units
	Min	Mean	Max	Min	Mean	Max	
Calcite concentration	0	0.01	0.04	0	0	0.06	mol/l
Maximum chlorophyll *a*	8.12	40.47	64.57	0.33	5.38	64.57	mg/m^3^
Mean chlorophyll *a*	8.12	29.46	47.81	0.22	3.04	53.65	mg/m^3^
Minimum chlorophyll *a*	3.35	20.54	32.91	0.09	1.55	41.41	mg/m^3^
Chlorophyll *a* range	0	26.2	35.47	0	3.83	53.63	mg/m^3^
Cloud cover maximum	0.79	0.84	0.9	0.65	0.88	0.98	%
Cloud cover mean	0.72	0.76	0.78	0.49	0.77	0.92	%
Cloud cover minimum	0.62	0.67	0.7	0.27	0.65	0.84	%
Dissolved oxygen	5.72	6.03	8.33	4.95	6.44	8.4	ml/l
Nitrate	1.06	19.57	27.62	0.48	3.42	46.18	μmol/l
Maximum photosynthetically available radiation	41.4	45.85	48.53	39.46	46.75	59.72	Einstein/m^2^/d
Mean photosynthetically available radiation	27.51	30.85	34.22	26.99	31.08	37.34	Einstein/m^2^/d
pH	8.18	8.21	8.24	7.54	8.18	8.37	–
Phosphate	0.14	1.03	1.24	0.04	0.35	2.26	μmol/l
Salinity	5.49	28.16	31.81	3.83	30.05	38.42	PSS
Silicate	8.89	14.81	18.57	0.4	6.02	25.23	μmol/l
Maximum SST	17.07	19.91	23.5	13.01	20.12	31.85	°C
Mean SST	6.57	11.41	12.77	5.13	12.01	19.9	°C
Minimum SST	−1.15	2.63	7.28	−1.5	5.63	13.85	°C
SST range	11.37	17.28	24.64	4.87	14.49	28.48	°C
**Inland**	**Min**	**Mean**	**Max**	**Min**	**Mean**	**Max**	**Units**
Maximum value of the daytime LST	19	25.45	39	11	31.51	54	°C
Minimum value of the daytime LST	−21	−6.04	3	−30	−11.13	9	°C
Mean value of the daytime LST	8	12.44	23	−5	13.86	33	°C
Maximum value of the nighttime LST	13	18.18	26	4	18.23	27	°C
Mean value of the nighttime LST	1	6.69	13	−8	3.13	16	°C
Minimum value of the nighttime LST	−29	−10.97	0	−38	−16.98	4	°C

Values based on known occurrences (observed environmental range) and those predicted by the ecological niche model (modeled environmental range).

**Table 3:** Bioclimatic variables used in this study.

Variable	Bioclim	Description	Unit
V1	Bio1	Annual mean temperature	°C
V2	Bio2	Mean diurnal range	°C
V3	Bio3	Isothermality	%
V4	Bio4	Temperature seasonality	%
V5	Bio5	Maximum temperature of the warmest month	°C
V6	Bio6	Minimum temperature of the coldest month	°C
V7	Bio7	Temperature annual range	°C
V8	Bio8	Mean temperature of the wettest quarter	°C
V9	Bio9	Mean temperature of the driest quarter	°C
V10	Bio10	Mean temperature of warmest quarter	°C
V11	Bio11	Mean temperature of coldest quarter	°C
V12	Bio12	Annual precipitation	mm/m^2^
V13	Bio13	Precipitation of the wettest month	mm/m^2^
V14	Bio14	Precipitation of the driest month	mm/m^2^
V15	Bio15	Precipitation seasonality	%
V16	Bio16	Precipitation of the wettest quarter	mm/m^2^
V17	Bio17	Precipitation of the driest quarter	mm/m^2^
V18	Bio18	Precipitation of warmest quarter	mm/m^2^
V19	Bio19	Precipitation of coldest quarter	mm/m^2^

**Table 4:** Remote sensing environmental variables used in this study.

Ocean	Units
Calcite concentration	mol/l
Maximum chlorophyll a	mg/m^3^
Mean chlorophyll a	mg/m^3^
Minimum chlorophyll a	mg/m^3^
Chlorophyll a range	mg/m^3^
Cloud cover maximum	%
Cloud cover mean	%
Cloud cover minimum	%
Dissolved oxygen	ml/l
Nitrate	μmol/l
Maximum photosynthetically available radiation	Einstein/m^2^/day
Mean photosynthetically available radiation	Einstein/m^2^/day
pH	–
Phosphate	μmol/l
Salinity	PSS
Silicate	μmol/l
Maximum SST	°C
Mean SST	°C
Minimum SST	°C
SST range	°C
**Land**	**Units**
Maximum value of daytime LST	°C
Minimum value of daytime LST	°C
Mean value of daytime LST	°C
Maximum value of nighttime LST	°C
Mean value of nighttime LST	°C
Minimum value of nighttime LST	°C

**Table 5:** The eigenvector coefficients of a standardized principal component analysis of original climatic variables.

No.	Climatic variable	Axis 1	Axis 2	Axis 3	Axis 4
V1	Annual mean temperature	0.268549	−0.30154	−0.04182	−0.06167
V2	Mean diurnal range	0.154837	−0.40671	0.153434	−0.0833
V3	Isothermality	0.296945	−0.19697	−0.16491	−0.0661
V4	Temperature seasonality	0.285549	0.178237	0.228065	0.026596
V5	Maximum temperature of the warmest month	0.27084	0.077764	0.233843	0.266797
V6	Minimum temperature of the coldest month	0.22525	0.235566	0.235308	−0.25582
V7	Temperature annual range	−0.02231	−0.28657	−0.05584	0.519083
V8	Mean temperature of the wettest quarter	0.268126	0.122484	0.232199	0.2296
V9	Mean temperature of the driest quarter	0.2369	0.228993	0.225927	−0.25839
V10	Mean temperature of the warmest quarter	0.16399	0.029729	0.409481	0.267514
V11	Mean temperature of the coldest quarter	0.264259	0.217343	0.02297	−0.1566
V12	Annual precipitation	−0.22286	−0.1774	0.317968	−0.22584
V13	Precipitation of the wettest month	−0.10389	−0.18329	0.143856	−0.44201
V14	Precipitation of the driest month	−0.26818	−0.03707	0.324793	0.041671
V15	Precipitation seasonality	0.065615	−0.42041	0.230196	−0.16373
V16	Precipitation of the wettest quarter	0.298626	−0.16991	−0.19483	−0.05618
V17	Precipitation of the driest quarter	−0.26579	−0.07492	0.334569	−0.03937
V18	Precipitation of the warmest quarter	0.139045	−0.33209	0.183675	0.184543
V19	Precipitation of the coldest quarter	0.262728	−0.161	−0.21275	−0.22105

Note: The eigenvalues of the first four axes are: axis 1 = 0.4453, axis 2 = 0.2112, axis 3 = 0.1651, and axis 4 = 0.0854 (sum = 90.73% of total variance explained).
