# Supplementary material for: Realized niche shift associated with the Eurasian charophyte Nitellopsis obtusa becoming invasive in North America
Source: Sci Rep. 2016 Jul 1;6:29037. doi: 10.1038/srep29037 (PMC4929560; doi:10.1038/srep29037)
Supplement: Supplementary Information [file srep29037-s2.pdf]

**Realized niche shift associated with the Eurasian charophyte *Nitellopsis obtusa* becoming invasive in North America**

**Authors**

Luis E. Escobar, Huijie Qiao, Nicholas B. D. Phelps, Carli K. Wagner, Daniel J. Larkin

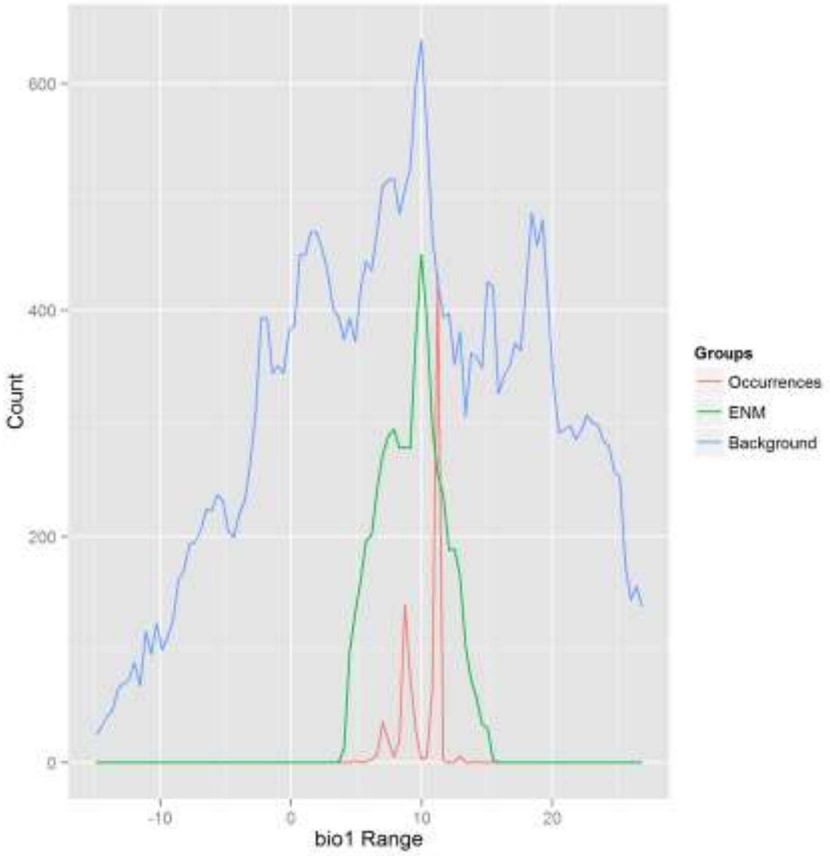


**Figures S2. Environmental distribution of *Nitellopsis obtusa* based on annual mean temperature (°C).** Lines represent actual occurrences (red), predictions based on the ecological niche model (green), and background values found in the study area (blue). Axis *x* is the environmental range of the variable, axis *y* represents the number of cells for each environmental value.

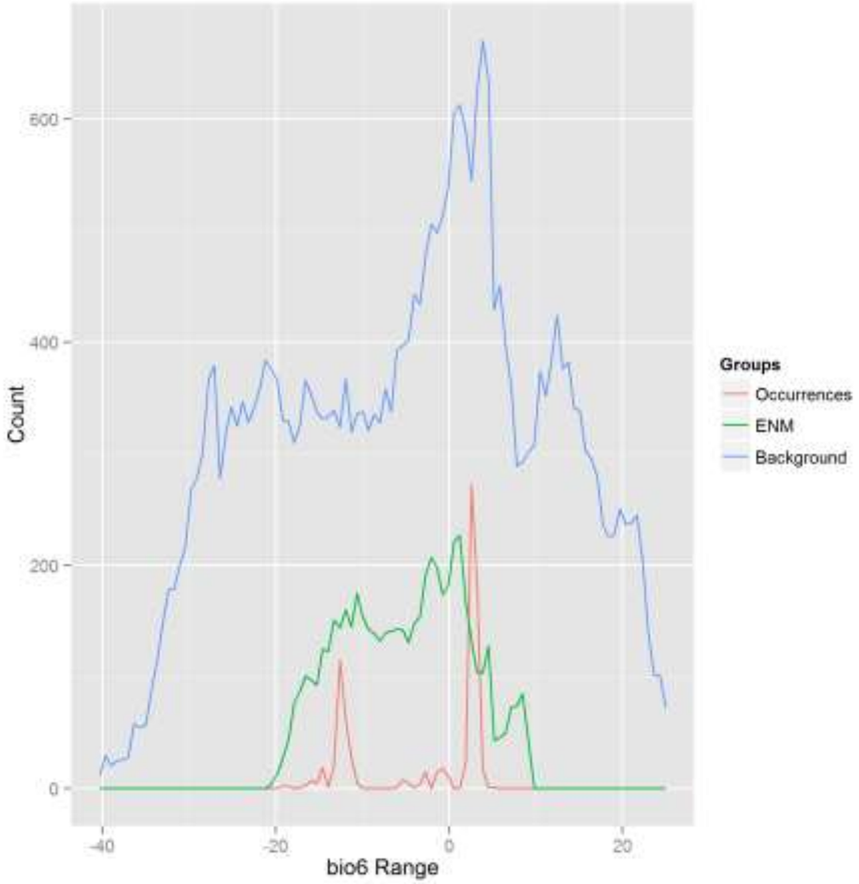


**Figures S3. Environmental distribution of *Nitellopsis obtusa* based on minimum temperature of coldest month (°C).** Lines represent actual occurrences (red), predictions based on the ecological niche model (green), and background values found in the study area (blue). Axis *x* is the environmental range of the variable, axis *y* represents the number of cells for each environmental value.

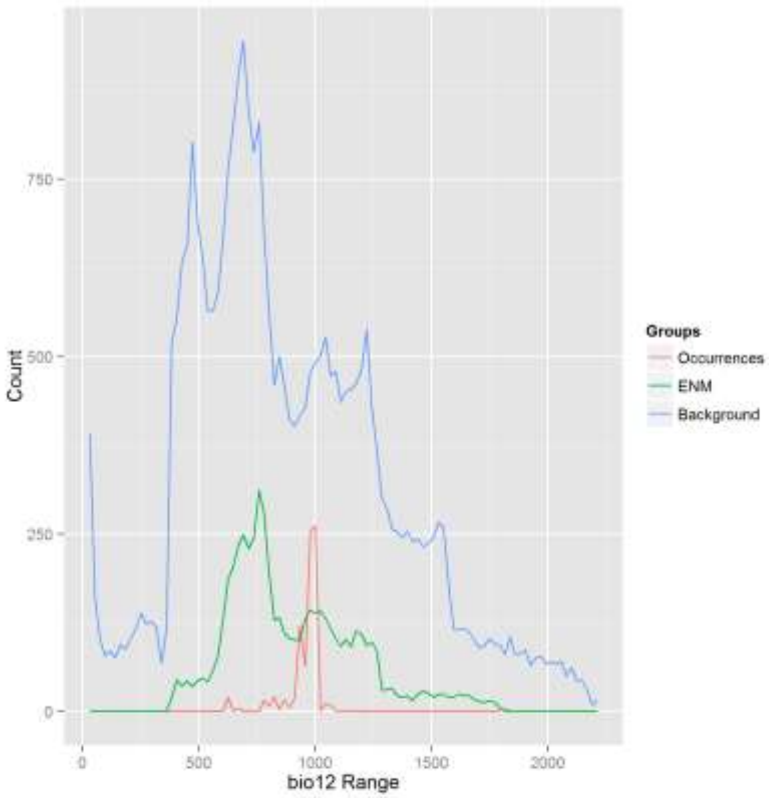


**Figures S4. Environmental distribution of *Nitellopsis obtusa* based on annual precipitation (mm/m$^2$).** Lines represent actual occurrences (red), predictions based on the ecological niche model (green), and background values found in the study area (blue). Axis *x* is the environmental range of the variable, axis *y* represents the number of cells for each environmental value.

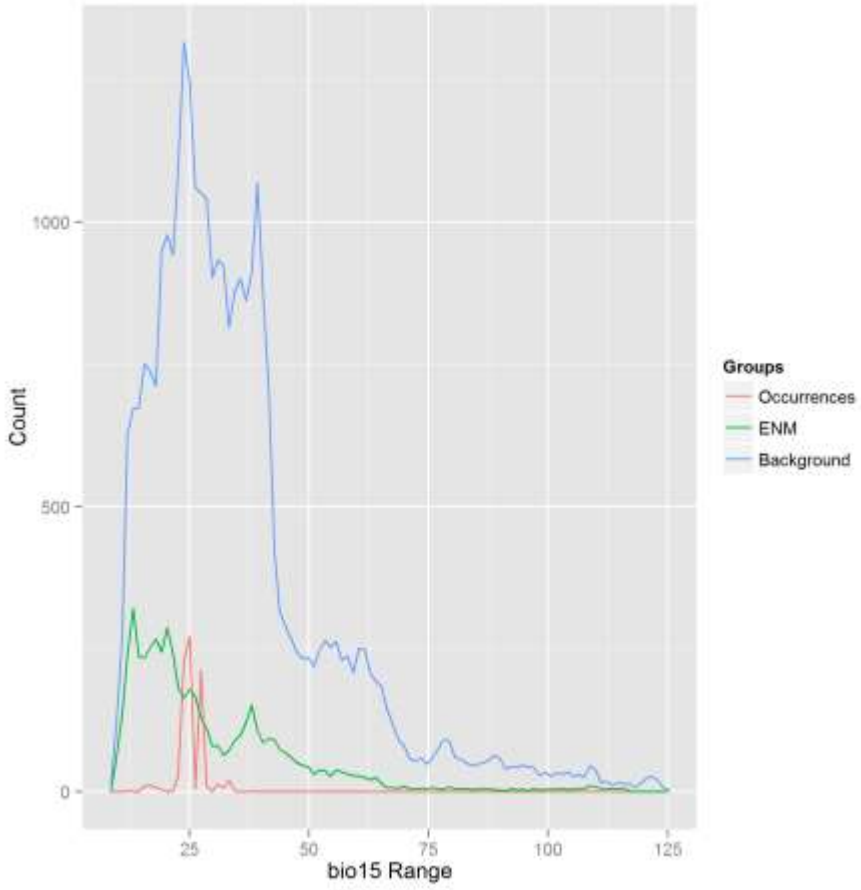


**Figures S5. Environmental distribution of *Nitellopsis obtusa* based on precipitation seasonality (%).** Lines represent actual occurrences (red), predictions based on the ecological niche model (green), and background values found in the study area (blue). Axis *x* is the environmental range of the variable, axis *y* represents the number of cells for each environmental value.

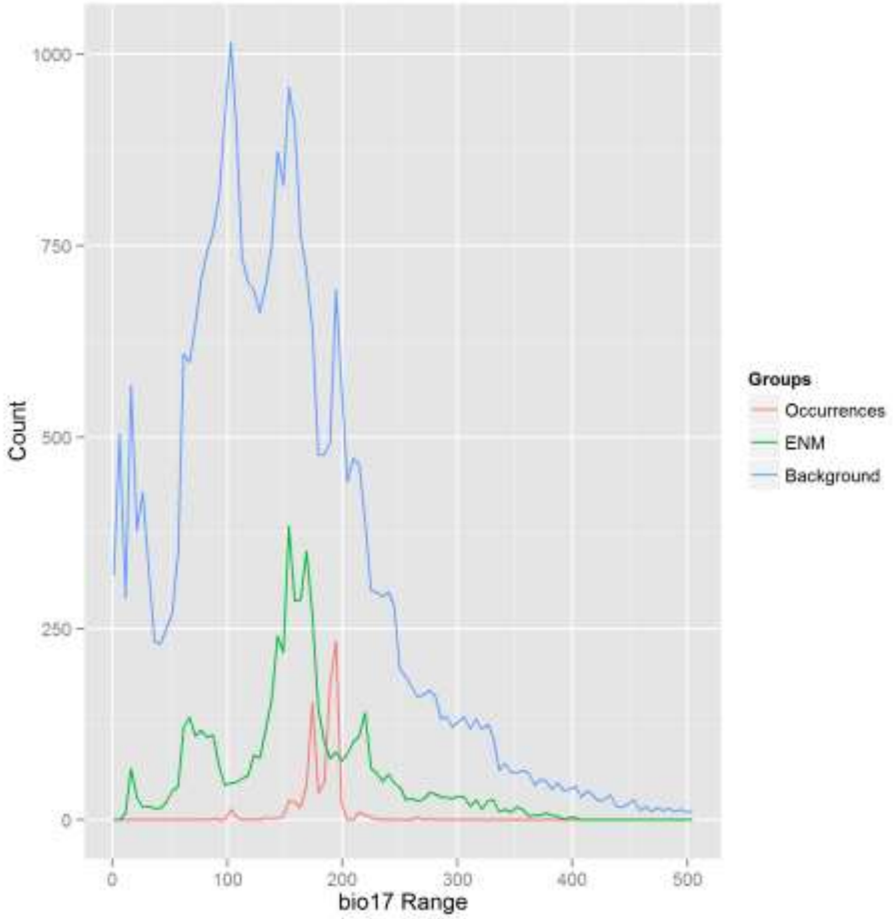


**Figures S6. Environmental distribution of *Nitellopsis obtusa* based on precipitation of driest quarter (mm/m$^2$).** Lines represent actual occurrences (red), predictions based on the ecological niche model (green), and background values found in the study area (blue). Axis *x* is the environmental range of the variable, axis *y* represents the number of cells for each environmental value.
